# Editorial Comment to Resumption of anti‐programmed cell death 1 monotherapy for severe immune‐related adverse events experienced patient with renal cell carcinoma

**DOI:** 10.1002/iju5.12181

**Published:** 2020-06-25

**Authors:** Ario Takeuchi, Masaki Shiota

**Affiliations:** ^1^ Department of Urology Graduate School of Medical Sciences Kyushu University Fukuoka Japan

In this issue, Maegawa *et al*.^1^ reported on the safety and efficacy of reinitiating nivolumab monotherapy in a case who discontinued combination therapy of nivolumab and ipilimumab due to immune‐related adverse events (irAEs). Although the development of immune‐checkpoint inhibitors (ICIs) has changed the treatment of advanced renal cell carcinoma, the clinical practitioners are confronted with a problem of whether ICIs should be reinitiated following recovery from severe irAEs.

So far, several case‐series studies on the incidence of irAEs with the resumption of ICIs after a discontinuation due to irAEs were reported. Santini *et al*. reported that 26% had recurrence of the initial irAEs, and 26% had new irAEs in advanced non‐small cell lung cancer.^2^ In the study by Pollack *et al*., 18% had recurrent irAEs, and 21% had distinct toxicities in metastatic melanoma.^3^ Simonaggio *et al*. showed that 42.5% and 12.5% had recurrence of the same and different irAEs in various cancers, respectively.^4^ Notably, two (pneumonitis, colitis) and one (Stevens–Johnson syndrome) treatment‐related deaths occurred after the resumption of anti‐PD‐(L)1.^2^, ^3^ Accordingly, great caution is required when ICIs are re‐administered after irAEs. Remaining on steroid therapy at resumption, and shorter time to initial irAEs were suggested to be associated with recurrent irAEs in resumption, which might be useful to estimate the risk of irAEs re‐emergence.^3^, ^4^

With regard to the efficacy of ICIs resumption, Santini *et al*. demonstrated favorable prognosis with the resumption of anti‐PD‐(L)1 although statistical power is not enough. In the study by Pollack *et al*., 31% had partial responses, 23% had stable disease, and 46% had progressive disease among patients underwent resumption of ICIs for disease progression after discontinuation.^3^ Simonaggio *et al*., 32.5% had a partial response, 37.5% had stable disease, and 22.5% had progressive disease during the resumption of ICIs.^4^ Collectively, data on the efficacy of resumption suggest a promise of ICI re‐challenge in selected patients.

Thus, re‐challenge of ICIs shows expected tumor response in exchange for higher risk of irAEs, which can rarely be lethal. In line with these previous reports, this case report demonstrated controllable irAE after resumption of nivolumab although objective response was not obtained. Since there is no established evidence supporting the usefulness of resumption of ICIs after irAEs, resumption of ICIs should be determined after adequate discussions with patients.

## Conflict of interest

The authors declare no conflict of interest.
